# Assessment of biosafety and implantation feasibility of novel phakic refractive lens

**DOI:** 10.1007/s10792-022-02345-y

**Published:** 2022-05-13

**Authors:** Shaohua Zhang, Chang Huang, Huamao Miao, Junyao Wu, Chao Xing, Zhaoxing Dai, Jianguo Sun

**Affiliations:** 1 Eye Institute and Department of Ophthalmology, Eye and ENT Hospital, Fudan University, Shanghai, 200031 China; 2 NHC Key Laboratory of Myopia (Fudan University), Shanghai, 200031 China; 3 Key Laboratory of Myopia, Chinese Academy of Medical Sciences, Shanghai, 200031 China; 4 Shanghai Key Laboratory of Visual Impairment and Restoration, Shanghai, 200031 China; 5 Hangzhou Aijinglun Technology Co., Ltd., Zhejiang, 311100 China; 6 Shanghai Haohai Biological Technology Co. Ltd., Shanghai, 200052 China

**Keywords:** Phakic refractive lens, Biosafety, Implantation feasibility, Circulation of aqueous humor, Intraocular pressure

## Abstract

**Purpose:**

We investigated the biosafety and implantation feasibility of a new phakic refractive lens (PRL) in rabbit eyes.

**Methods:**

Short PRLs (S-PRLs), large PRLs (L-PRLs), and large-grooved PRLs (LG-PRLs), were prepared by molding medical-grade liquid silicon. The cytotoxicity and cellular adhesion of the PRLs was assessed in vitro. To assess implantation feasibility, the S-PRL, L-PRL, and LG-PRL were implanted in the posterior chamber of rabbit eyes and the relative position was assessed by optical coherence tomography. The intraocular pressures (IOP) were compared between the S-PRL, L-PRL, LG-PRL, and control groups to evaluate the PRL biosafety after implantation.

**Results:**

The in vitro assays showed that cell viability and cellular adhesion in the S-PRL, L-PRL and LG-PRL groups was not significantly different to those in the control group throughout the study. After implantation into the posterior chamber of rabbit eyes, there were no obvious signs of inflammation or increases in IOP at each time point relative to the control group, demonstrating good biosafety of the PRL. The relative positions of the L-PRLs and LG-PRLs in the posterior chamber were appropriate and the retention frequencies were high.

**Conclusions:**

The newly developed LG-PRL showed good biosafety with negligible in vitro cytotoxicity, ocular inflammation, or fluctuations in IOP. The LG-PRL provided the best implantation feasibility. The grooves on the LG-PRL provided channels for aqueous humor circulation. The LG-PRL is a promising type of PRL with an appropriate size and surface structure for effective correction of refractive errors in rabbit eyes.

## Introduction

Myopia is one of the most common eye diseases worldwide [1], affecting 10–30% of adults in many countries and 80–90% of young adults in some parts of East and Southeast Asia [2]. The effect of optical interventions, and pharmaceutical and behavioral modifications to delay the onset and progression of myopia have been studied [3]. Optical interventions include spectacles, contact lenses, corneal laser refractive surgery, refractive lens exchange, and phakic intraocular lens (pIOL) implantation [4].

Implantation of a pIOL can preserve the accommodative visual function and is appealing to surgeons and young patients [5]. There are currently two types of pIOLs: implantable collamer lenses (ICLs) and phakic refractive lenses (PRLs). ICLs are the most widely used owing to their proven safety and effectiveness [6]. Supplemental laser or surgical iridectomy is often performed to preserve aqueous flow from the posterior to the anterior chamber, although this increases the treatment time, cost, and risk of complications [7]. To address this problem, a centrally perforated ICL has been developed, which improves aqueous humor drainage and had good implantation safety and efficacy, reducing the need for iridectomy [8]. A third-generation PRL composed of hydrophobic silicon with the same density as aqueous humor has also been developed [9]. This type of PRL is suspended in the space between the iris and the lens [9], and does not apply pressure to the ciliary structure or the anterior surface of the crystalline lens. However, there are potential complications associated with PRL implantation, including pigmentary glaucoma, secondary cataract, postsurgical flare, and traumatic aniridia [9]; these complications may be related to the shape of the PRL and the implantation location.

**Fig. 1:**
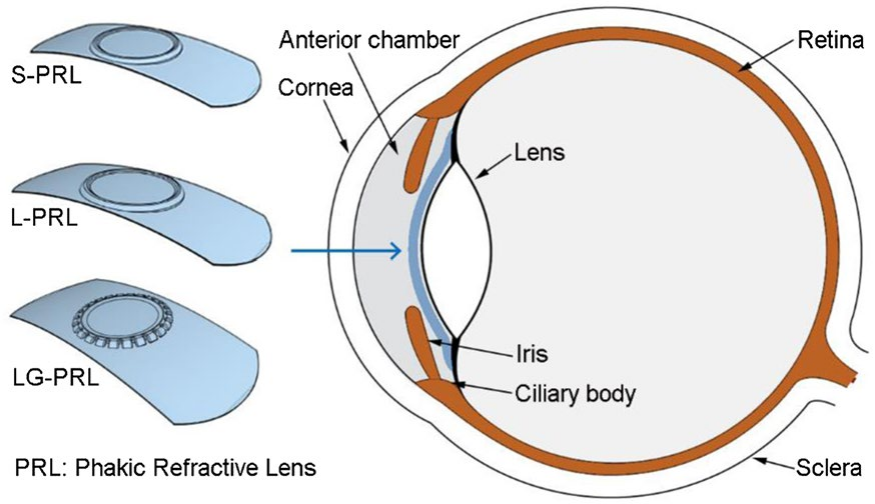
Designs of the three types of PRL and intraocular implantation

Considering this background, we designed and prepared a new PRL with grooves surrounding the central optical zone (large-grooved PRL [LG-PRL]) to improve aqueous humor drainage and reduce the risk of ocular complications. This PRL is implanted in the posterior chamber and occupies the slit potential space between the posterior surface of the iris and the anterior surface of the crystalline lens. The design of the LG-PRL and its implantation location are shown in Fig. 1. To investigate the biosafety and implantation feasibility of the LG-PRL in rabbit eyes, we also prepared a short PRL (S-PRL) and a large PRL (L-PRL). We evaluated the biosafety of the three types of PRL in terms of in vitro cytotoxicity, in vivo ocular inflammation, and fluctuations in intraocular pressure (IOP). The implantation feasibility was evaluated by observing the relative position, calculating the retention frequency in the posterior chamber of rabbit eyes, and evaluating the available space for aqueous humor circulation.

## Materials and methods

### Materials

We designed three types of PRL with different lengths and features: S-PRL (12.3 mm long), L-PRL (13.3 mm long), and LG-PRL (13.3 mm long with grooves surrounding the central optical zone). The S-PRLs are exactly the same with those widely examined by clinical application [9], while the L-PRLs and LG-PRLs are specially designed for rabbits according parameters listed below. We tried to use slit-lamp examination, UBM and anterior OCT for measurements, but these devices are not designed for rabbits, so accurate measurement is not feasible. Thus, we mainly referred to the literature [10] (Tables 1, 2).

**Table 1:** Parameters of rabbit eyes

Structures	Parameters	Size (mm)
Cornea	Horizontal diameter	15.0
	Vertical diameter	13.5–14.0
	Radius of curvature	7.0–7.5
Pupil diameter	Light pupil	5.0
	Dark pupil	7.0
Anterior chamber depth	Anterior chamber depth	2.9 ± 0.36
Lenes	Curvature radius of posterior surface	5.0
	Curvature radius of front surface	5.3
	Equatorial diameter	9.0–11.0
	Thickness	7.0

**Table 2:** Parameters of three groups of PRLs

Parameters	S-PRL	L-PRL	LG-PRL
Length	12.3 mm	13.3 mm	13.3 mm
Width	6.0 mm	6.0 mm	6.1 mm
Radius of curvature of rear surface	9.6 mm		
Optical zone diameter	5.0 mm	5.0 mm	5.6 mm
Diopter	− 14.5 D		

According to the above parameters, we designed the PRLs with following sizes and implanted them into rabbit eyes.

All PRL samples were sterilized by UV irradiation at 254 nm for 40 min. Eagle’s minimum essential medium (EMEM) and fetal bovine serum (FBS) were obtained from Scientific Lab (Shanghai, China). Penicillin (100 units/mL) and streptomycin (0.1 mg/mL) were bought from Sigma-Aldrich Co., Ltd. (St. Louis, MO, USA). The cell counting kit-8 (CCK-8) assay kit was bought from Dojindo Co., Ltd. (Kumamoto, Japan). All other chemicals were of analytical grade and used without further purification. Other chemicals, drugs or materials included ofloxacin eye ointment (Shenyang Sinqi, Shenyang, China), 0.1% betamethasone (Rinderon; Shionogi, Osaka, Japan), 0.5% levofloxacin (Cravit; Santen, Osaka, Japan), topicamide eye drops (Alcon, Beijing, China), oxybucaine hydrochloride eye drops (Santen), and pilocarpine eye drops (Bausch + Lomb, Laval, Canada).

### Characterization of the PRLs

Digital photographs of the S-PRL, L-PRL, and LG-PRL were taken with a camera (CANON EOS 60D, Canon, Tokyo, Japan). The transparency of each PRL was measured using an ultraviolet–visible (UV–vis) light spectrophotometer (Biomate 3S, Thermo Fisher Scientific, Waltham, MA, USA) with a scanning wavelength of 250–800 nm.

### In vitro cytotoxicity and cell adhesion

A human lens epithelial cell line (HLEC, SRA01/04) was obtained from Shanghai Genechem Inc. (Shanghai, China). The HLECs were cultured in EMEM supplemented with FBS (10%), penicillin (100 units/mL), and streptomycin (0.1 mg/mL). The cells were incubated at 37 °C in a humidified atmosphere of 5% CO_2_. The HLECs were seeded onto 24‐well plates at a density of 1 × 10^5^ cells per well and incubated at 37 °C for 24 h. A PRL was placed in a Corning Transwell membrane insert (Sigma-Aldrich) and cultured with the HLECs for 24 or 48 h. The CCK-8 assay kit was used to measure in vitro cytotoxicity by measuring absorbance at 450 nm with a microplate reader, BioTek, Winooski, VT, USA). The results were calculated as the cell viability as a percentage of that in the control group of cells incubated in the well without a PRL.

To assess cellular adhesion, pieces of PRL film (1.5 mm diameter) were placed into the wells of a 24-well plate and fixed with a polytetrafluoroethylene ring. HLECs were then seeded into wells containing the PRL film at a density of 1 × 10^5^ cells per well. The plates were then incubated at 37 °C for 24 or 48 h. The HLECs that adhered to the surface of the PRL film were stained with calcein-acetoxymethyl and fluorescent images were acquired by fluorescent microscopy.

### Intraocular implantation of the PRLs

Male New Zealand white rabbits (2.5 kg) were housed individually in a light-controlled room at 20 ± 1 °C with free access to food and water. A total of 13 rabbits (26 eyes) were used for the in vivo study, including 8 eyes for the LG-PRL group, 8 eyes for L-PRL, 6 eyes for S-PRL, and 4 eyes for blank control. Rabbits were grouped using a randomized block design. Experiments began after 2 weeks of acclimatization to the animal room. All animal experiments were approved by the Animal Ethics Committee of the Eye and ENT Hospital of Fudan University (Shanghai, China), and all experimental protocols, including care, transportation and experiments of the animals, complied with the guidelines of the Animal Care and Use Committee of Fudan University and the Association for Research in Vision and Ophthalmology Statement for the Use of Animals in Ophthalmic and Vision Research.

Topicamide eye drops and oxybucaine hydrochloride eye drops were topically administered before surgery. After placing an ophthalmic viscosurgical device (OVD; Opegan, Santen, Osaka, Japan) into the anterior chamber, a S-PRL, L-PRL, or LG-PRL was implanted in the posterior chamber through a 3-mm clear corneal incision using an injector cartridge. The OVD was washed out with balanced salt solution, and pilocarpine eye drops were topically administered. Postoperatively, 0.1% betamethasone and 0.5% levofloxacin eyedrops were topically administered four times daily for 2 weeks. All surgical procedures were performed by the same experienced surgeon. Slit-lamp biomicroscopy was performed before surgery and at 1, 3, 5, 7, 10, and 15 days postoperatively in each eye. IOP was measured using a handheld digital tonometer (Tono-Pen XL, Reichert Inc., Depew, NY, USA) under general and local anesthesia, as described above. Ocular inflammation and the relative location of the PRLs were observed by normal and slit-lamp examination at 1, 3, 5, 7, 10, and 15 days postoperatively. The relative positions of the PRLs in the posterior chamber were confirmed by optical coherence tomography (OCT).

### Statistical analysis

All experiments were conducted in triplicate, and data are expressed as the mean ± standard deviation. Independent-sample *t*-tests were used to compare in vitro cytotoxicity and cell adhesion. One-way analysis of variance was used to compare IOP among the study groups. The *χ*^2^ test was used to compare the retention frequency between the three types of PRL. Statistically significant differences were defined by a confidence level of 95% (*p* < 0.05).

## Results

### Characterization of the PRLs

**Fig. 2:**
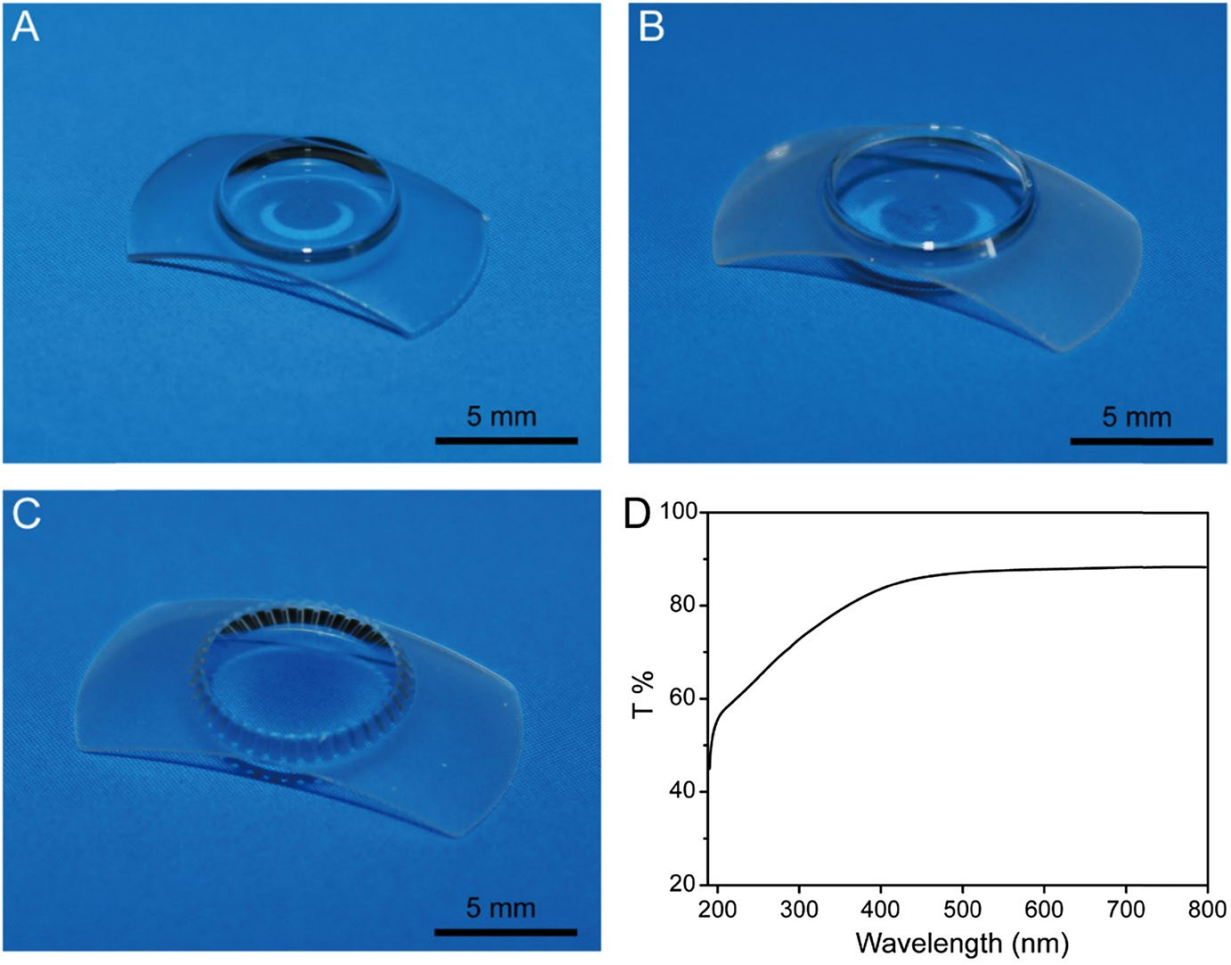
Representative photographs of the S-PRL (A), L-PRL (B), and LG-PRL (C), and UV–vis light transmittance (D)

The S-PRLs, L-PRLs, and LG-PRLs were prepared by molding medical-grade liquid silicon. Photographs of representative PRLs are shown in Fig. 2A–C.

Figure 2D shows the light transmittance curve of the PRL. Visible light transmittance through the PRL optic column exceeded 90% for wavelength of 500–700 nm, consistent with the measurements obtained in a previous report [11] and approximated that of the natural cornea [12]. The PRL optic column blocked UV light at wavelengths of 350–400 nm, which may reduce the damaging effects of UV light on internal eye tissues.

### In vitro cell cytotoxicity and cell adhesion

**Fig. 3:**
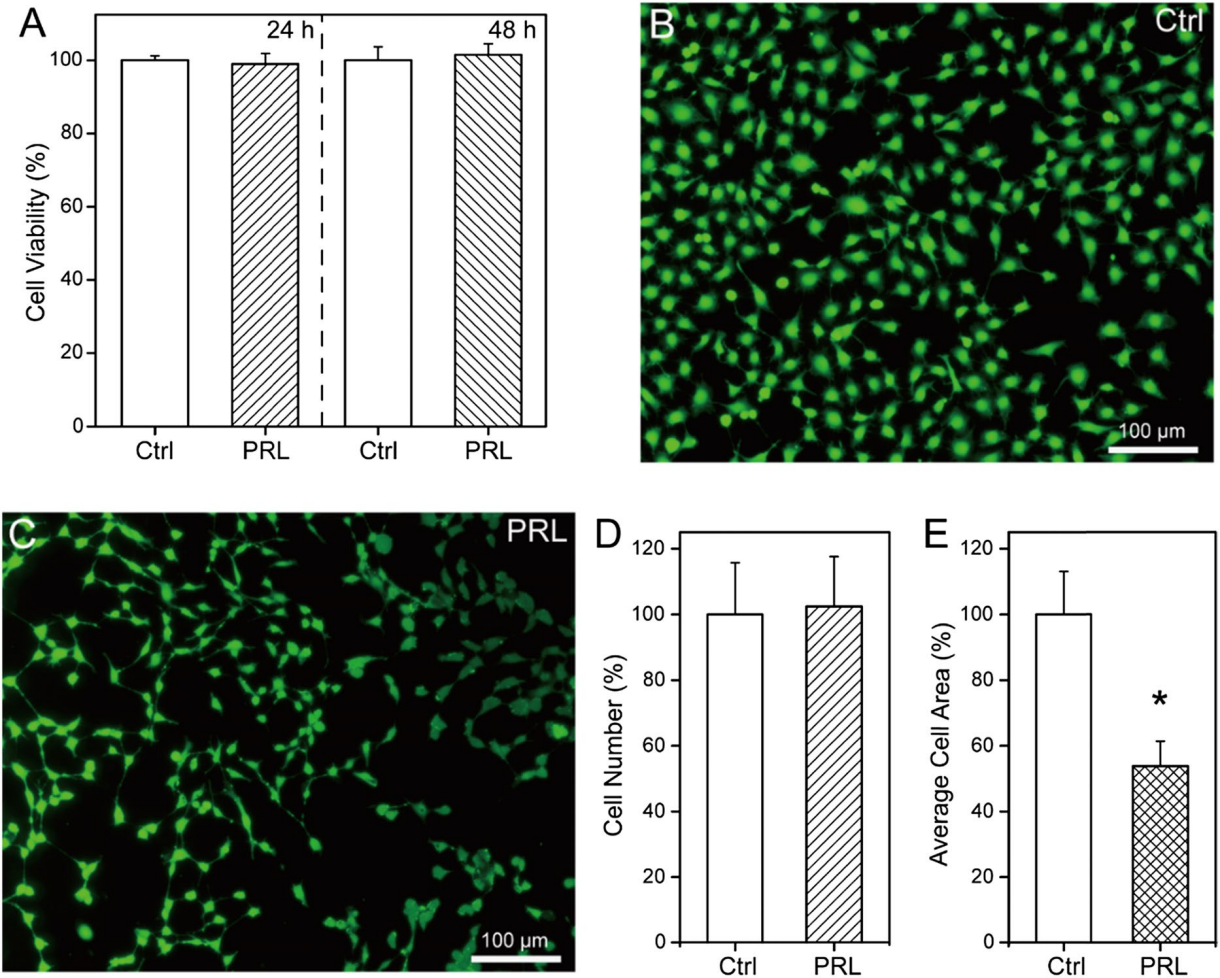
**A** In vitro cell cytotoxicity of HLECs cultured on the PRL surface for 24 or 48 h. **B**, **C** Representative photographs showing morphology of adherent HLECs on the control (**B**) and PRL surface **C** in 12-well plates at 24 h. **D**, **E** Cell count (**D**) and average cell area **E** of HLECs adhered to the control well and PRL surface

The in vitro cytotoxicity of the PRL was investigated by incubating them with HLECs and cell viability was measured using a CCK-8 assay. The results are shown in Fig. 3A. The viability of HLECs cultured with the PRL for 24 or 48 h was not significantly different from that in the control group.

Figure 3B, C show the adhesion of HLECs to the well surface in the control group and the PRL surface. The HLECs had a typical epithelial cell cobblestone-like shape, with long spindle-like cellular extensions, and extensive spread across the surface of the well (Fig. 3B). By contrast, when cultured on the PRL surface, the HLECs formed smaller aggregates with fewer cellular extensions. The cell count and average cell area were calculated to determine the adhesion of HLECs to the PRL surface. Intriguingly, the cell count was similar in both experimental conditions (Fig. 3D). However, the average cell area was significantly smaller in the PRL group than that in the control group (53.8% vs 100%, *p* < 0.05, Fig. 3E). This might be due to the weaker adhesion of HLECs to the soft PRL surface, consistent with findings of a previous report [13].

### Intraocular implantation of the PRLs

**Fig. 4:**
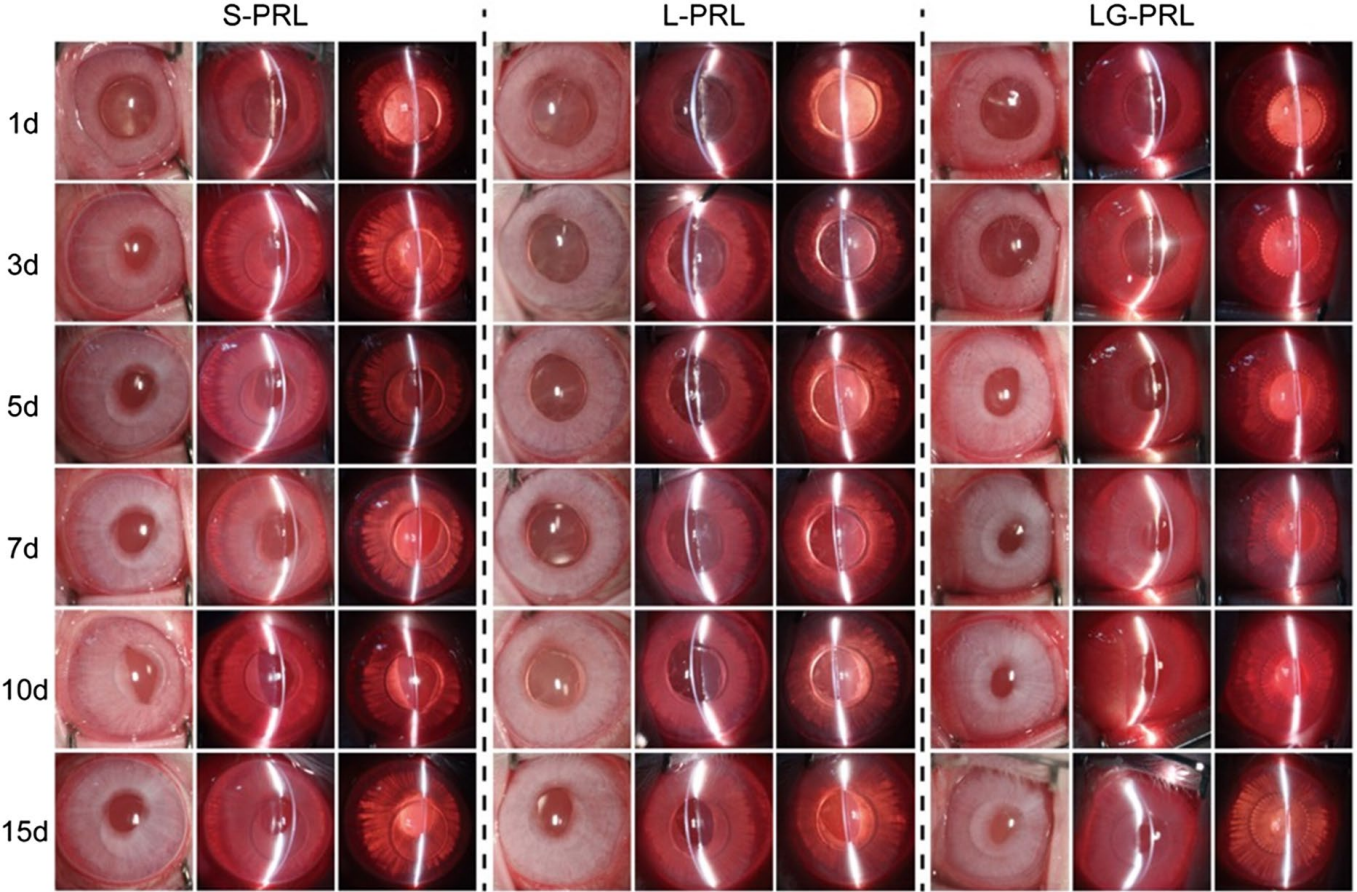
Representative photographs of the rabbit eyes after implantation of the PRLs. Photographs of the ocular surfaces (columns 1, 4, and 7; taken with diffuse illumination), anterior chamber (columns 2, 5, and 8; taken with direct focal illumination), and posterior chamber (columns 3, 6, and 9; taken with retro illumination) were taken in the normal and slit-lamp modes at 1, 3, 5, 7, 10, and 15 days after intraocular implantation of a S-PRL, L-PRL, or LG-PRL

After intraocular implantation of the three types of PRLs in rabbit eyes, we assessed ocular inflammation reactions and the relative location of the implanted PRL in each rabbit. Figure 4 shows representative photos of the ocular surfaces (columns 1, 4, and 7), anterior chamber (columns 2, 5, and 8), and the posterior chamber (columns 3, 6, and 9). Anterior chamber inflammation was visible at postoperative days 1 and 3 in all groups, but subsided by day 5 in all three groups. No serious complications, such as corneal opacity, keratopathy, or posterior synechia, were observed over 15 days in any rabbit.

**Fig. 5:**
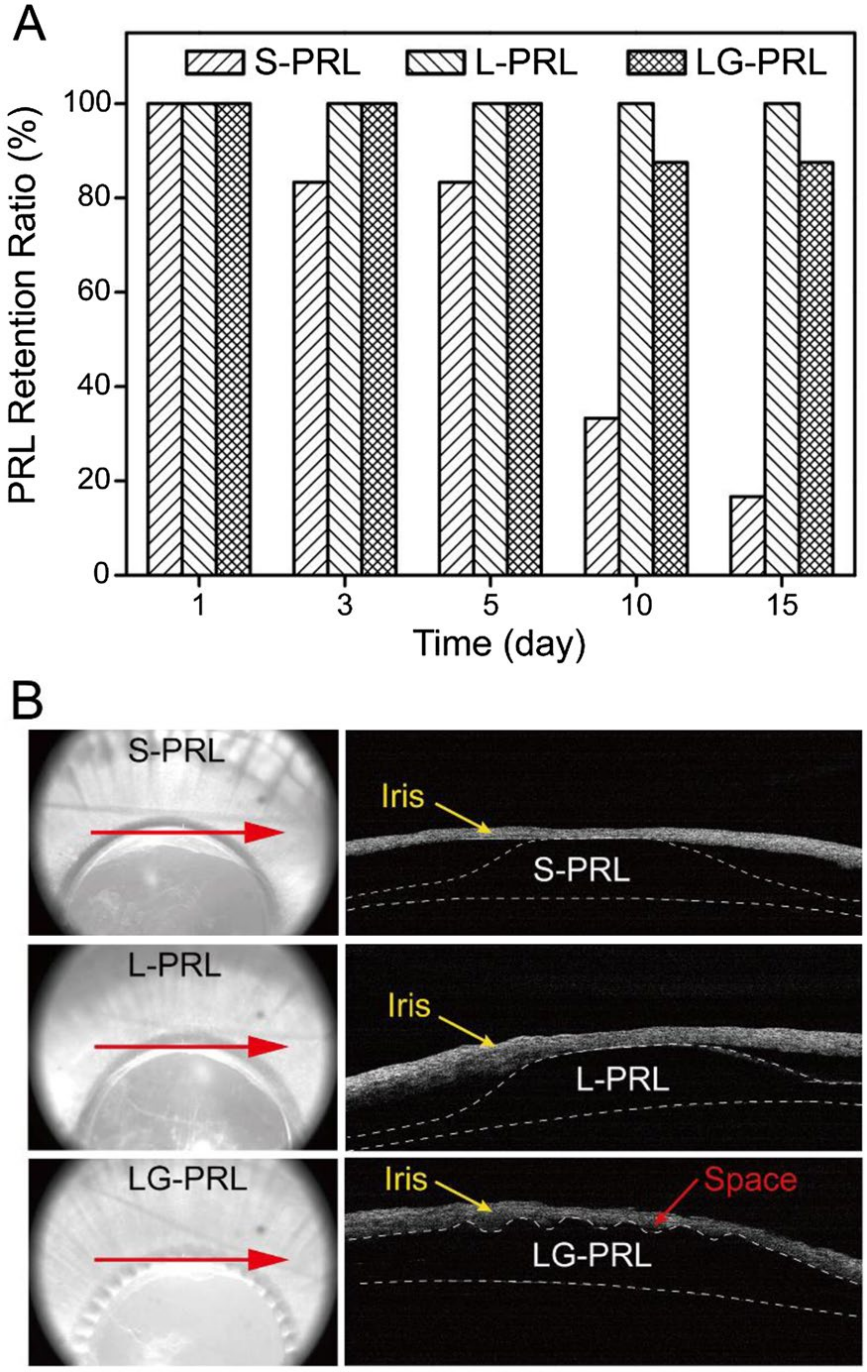
Retention frequency (**A**) and the relative position assessed by OCT (**B**) of the S-PRL, L-PRL, and LG-PRL following implantation in the posterior chamber. In **B**, the red arrows in the left-hand images show the direction of observation and location. The internal structures are shown in the right-hand images, which depict the relative positions and the available space between the implanted PRL and the iris

As indicated in Fig. 5A, all of the implanted S-PRLs were located in the posterior chamber at day 1. However, one S-PRL prolapsed into the anterior chamber and its dislocation was detected on day 3. Thereafter, two, one, and one additional S-PRLs were dislocated on days 7, 10, and 15. Therefore, the retention frequency for the S-PRLs was 16.7% at the final observation time (i.e., day 15). In comparison, all of the implanted L-PRLs were centered and stably located in the posterior chamber, with a retention frequency of 100%. One LG-PRL was dislocated on day 10, resulting in a retention frequency of 87.5%. The relative positions of the S-PRLs, L-PRLs, and LG-PRLs implanted in the posterior chamber were also assessed by OCT and the results are shown in Fig. 5B. OCT revealed that the implanted L-PRLs and S-PRLs were located in the posterior chamber and attached to the iris surface. By contrast, the LG-PRLs, which has grooves surrounding the central optical zone, left some gaps between the PRL and the iris surface. These gaps provided a channel for aqueous humor to drain from the posterior chamber to anterior chamber.

**Fig. 6:**
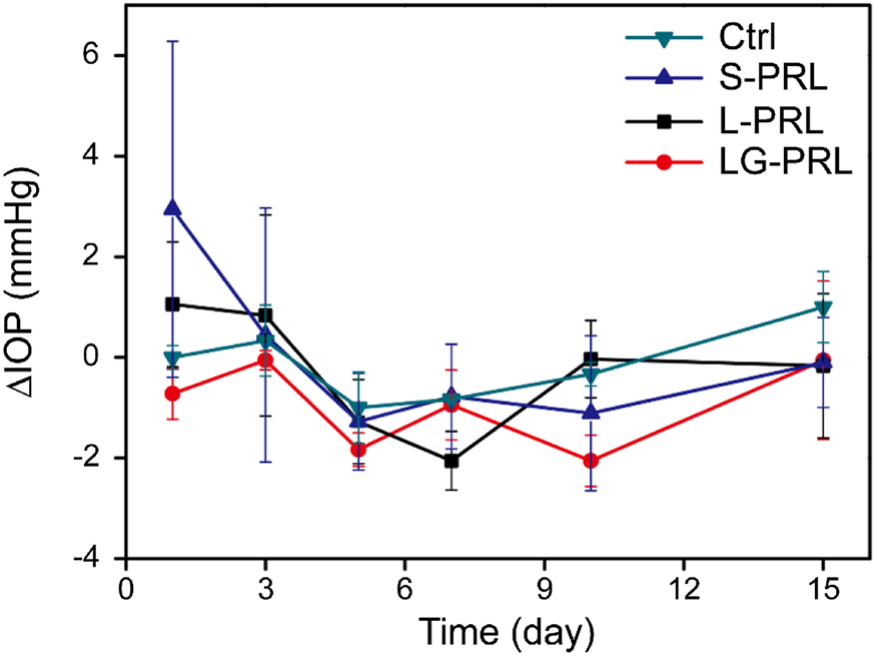
Postoperative changes in IOP (∆IOP) in the S-PRL, L-PRL, LG-PRL, and control groups at postoperative days 1, 3, 5, 7, 10, and 15

IOP was regularly measured during the experimental period and the results are shown in Fig. 6. The change in IOP (∆IOP) was used as a rough reference index for ocular safety in this normotensive model. The control group (normal eyes) had a relatively stable IOP (~ 11 mmHg), which was set as a baseline. ∆IOP was calculated as the difference in IOP between the baseline value and the values recorded in in the PRL groups. ∆IOP decreased slightly in all groups from day 3, but then increased between days 10 and 15. There were no significant differences in ∆IOP among the S-PRL, L-PRL, and LG-PRL groups compared to the control group at any time point.

## Discussion

Surgical correction of high myopia is a controversial issue and there are still no completely satisfactory techniques [14]. The predominant surgical method for treating high myopia is corneal refractive surgery [15], which has an excellent corrective effect, highly predictable outcomes, a low complication rate, and rapid visual recovery [16]. However, it is not feasible in myopic patients with a thin cornea or highly myopic patients if their residual stromal bed is too thin. Implantation of a pIOL (ICL or PRL) in individuals with high myopia has attracted widespread interest because it is a reversible and stable process that does not affect visual accommodation [17]. An ICL (V4c) has been developed with a central hole that allows adequate circulation of aqueous humor to maintain the normal condition of the anterior segment [18]. This ICL offers better efficacy and safety with a lower incidence of secondary cataracts than earlier types [19]. Nevertheless, the currently available PRL may still cause complications, which must be addressed to improve the outcomes of PRL implantation [9].

Hence, we designed and prepared three types of PRLs (S-PRLs, L-PRLs, and LG-PRLs) of different sizes or the presence of grooves to improve the outcomes by improving aqueous humor circulation. LG-PRL, a type of fluted PRL, is suitable for patients who are sensitive to pupillary block after PRL implantation. Our LG-PRL is designed with grooves surrounding the optical area to provide a channel for the circulation of aqueous humor, and their appropriate size which provides implantation stability (optical quality) and patient comfort. The PRL was optically very good with a UV–vis light transmittance of > 90% at wavelengths from 500 to 700 nm. The PRL material (hydrophobic silicon) showed negligible in vitro cytotoxicity and good biocompatibility when cultured with HLECs. Low adhesion of HLECs to intraocular implants is a key parameter for the success of intraocular surgery [20]. The PRL inhibited HLEC adhesion (Fig. 3), and thus is a promising intraocular implant. After intraocular implantation of the PRLs in rabbit eyes, no serious complications, such as corneal opacity, keratopathy, or posterior synechia, were observed over 15 days. The L-PRLs and S-PRLs attached to the iris surface after implantation, whereas the grooves in the LG-PRLs left channels across the iris surface for the circulation of aqueous humor. These findings indicate that the LG-PRL is more suitable than the S-PRL and L-PRL in rabbit eyes because it improves aqueous humor circulation and reduces postoperative complications.

IOP is an important index of the safety of PRL implantation. An elevated IOP after PRL surgery may be associated with common postoperative events, such as retention of viscoelastics or steroid responses [21, 22]. The IOP was regularly measured in each group and ∆IOP was calculated. ∆IOP was not significantly different among the S-PRL, L-PRL, LG-PRL, and control groups at each time point, indicating a low risk of glaucoma after PRL implantation. Overall, these findings suggest that intraocular implantation of the newly developed PRLs is safe and predictable, with long-term stability.

The retention frequency of LG-PRL (87.5%) was lower than that of L-PRL (100%) due to the dislocation of one LG-PRL, the reason for which was unclear. Accordingly, more work may be necessary to improve the implantation stability of LG-PRL in future. Moreover, studies with optical quality and visual performance should be carried out to determine the long-term safety and efficacy of this intraocular lens implant.

This study definitely has limitations. The main limitation of the present study is the small number of animals used in the study. We are currently conducting further research on more species, such as canine, gray rabbit or porcine models. More confirmatory results will be obtained in the follow-up studies. Also, we did not include a group of rabbits with no PRL implantation. This is a product that has been widely applied in clinical treatment, as mentioned above. Its materials have been proven in clinic for several years. So in this paper, we have mainly focused on the verification of the shape design rather than the material safety. Thirdly, we did not asses the rotation stability of PRLs. Indeed, any implants suspended in the posterior chamber have the possibility of rotation. And we did observe the PRL rotating from horizontal alignment to vertical 2 months after implantation clinically. But according to the long-term follow-up clinical observation, rotation of PRL has no influence on the biosafety and visual function [9, 23].

## Conclusions

In this study, we designed and prepared the S-PRL, L-PRL, and LG-PRL. These PRLs showed good biosafety with negligible in vitro cytotoxicity, ocular inflammation, and IOP fluctuations. Of the three types of PRLs, the LG-PRL, which provides channels for the circulation of aqueous humor, provided the best implantation feasibility with a high retention frequency in the posterior chamber of rabbit eyes. We believe that the LG-PRL is a promising alternative to conventional PRLs, with an appropriate size and surface structure, for the effective treatment of moderate to high refractive error.

## Data Availability

The data supporting the findings of this study are available within the article.
